# Replicon Typing of Plasmids Encoding Resistance to Newer β-Lactams

**DOI:** 10.3201/eid1207.051555

**Published:** 2006-07

**Authors:** Alessandra Carattoli, Vivi Miriagou, Alessia Bertini, Alexandra Loli, Celine Colinon, Laura Villa, Jean M. Whichard, Gian Maria Rossolini

**Affiliations:** * Istituto Superiore di Sanità, Rome, Italy;; † Institute Pasteur, Athens, Greece;; ‡ Università di Siena, Siena, Italy;; § Centers for Disease Control and Prevention, Atlanta, Georgia, USA

**Keywords:** β-Lactam resistance, plasmid, replicon typing, dispatch

## Abstract

Polymerase chain reaction–based replicon typing represents a novel method to describe the dissemination and follow the evolution of resistance plasmids. We used this approach to study 26 epidemiologically unrelated *Enterobacteriaceae* and demonstrate the dominance of incompatibility (Inc) A/C or Inc N-related plasmids carrying some emerging resistance determinants to extended-spectrum cephalosporins and carbapenems.

Understanding the molecular epidemiology of resistance plasmids has been a major issue since scientists became aware of plasmids' role in the spread of antimicrobial drug resistance. However, understanding this epidemiology has been complex because of the diversity and promiscuity of these elements. The plasmid replication system, which dictates the plasmid's behavior (host range, copy number) is the major plasmid landmark from a biologic standpoint; it is used for plasmid classification and identification (*1*). Plasmids were originally classified in incompatibility (Inc) groups (*2*). Inc is a manifestation of plasmid relatedness based on commonality of replication controls. The standard procedure for determining Inc groups requires laborious hands-on work, multiple conjugation, transformation assays, or hybridization experiments (*1**–**3*).

Our objective of understanding the relationship among resistance plasmids prompted us to develop a polymerase chain reaction (PCR)–based replicon typing method (*4*). Our study has 2 aims: 1) to investigate phylogenetic relatedness among plasmids carrying extended-spectrum cephalosporin (ESC) and carbapenem resistance determinants emerging in 3 different countries (Greece, Italy, and the United States) and 2) to ascertain the sensitivity of the method.

## The Study

PCR-based replicon typing was applied to type the resistance plasmids carried by 26 *Escherichia coli* transconjugants or transformants obtained from epidemiologically unrelated clinical isolates of *Enterobacteriaceae* associated with community- or hospital-acquired infections in the United States or southern Europe (Italy and Greece). The resistance plasmids carried genes encoding β-lactamases of Ambler class A (SHV-12), B (VIM-1 or VIM-4), and C (CMY-2, CMY-4, or CMY-13) (Table), which represent key emerging resistance determinants to ESC and carbapenems.

**Table:** Phenotypic and genetic characteristics of plasmids and transformant/transconjugant strains analyzed in this study

Original strain	Species and serovar	Transferred resistance traits in transconjugants or transformants*	*bla* genes identified on transferred plasmids	Replicons detected by PCR†
USA-4204	*Salmonella enterica* serovar Typhimurium	AmpCazCroCtxFox	CMY-2-type A	A/C_2_
USA-2039	*S.* Typhimurium	AmpCazCroCtxFoxGmTo	CMY-2-type A	A/C_2_
USA-3977	*S.* Typhimurium	AmpCazCroCtxFox	CMY-2-type A	A/C_2_
USA-8401	*Escherichia coli* O157:H7	AmpCazCroCtxFox	CMY-2-typeA	A/C_2_
USA-8749	*E. coli* O157:H7	AmpCazCroCtxFox	CMY-2-type A	A/C_2_
USA-8868	*E. coli* O157:H7	AmpCazCroCtxFox	CMY-2-type A	A/C_2_
USA-1091	*E. coli* O157:H7	AmpCazCroCtxFox	CMY-2-type A	A/C_2_
USA-7546	*E. coli* O157:H7	AmpCazCroCtxFox	CMY-2-type A	A/C_2_
USA-11371	*E. coli* O157:H7	AmpAtmCazCroCtxFox	CMY-2-type B	I1
USA-1358	*S.* Thompson	AmpAtmCazCroCtxFox	CMY-2-type B	I1
IT-VA416/02	*Klebsiella pneumoniae*	AmpAtmCazCroCtxFoxIpmGmTo	VIM-4, CMY-4	A/C_2_
IT-VA417/02	*Enterobacter cloacae*	AmpAtmCazCroCtxFoxIpmGmTo	VIM-4, CMY-4	A/C_2_
IT-FI045T	*Enterobacter aerogenes*	AmpAtmCaz	SHV-12	FII
IT-FI008T	*E. coli*	AmpAtmCazTo	SHV-12	FII
IT-BG003T	*Serratia marcescens*	AmpAtmCazTo	SHV-12	FII
IT-NO003T	*Klebsiella oxytoca*	AmpAtmCaz	SHV-12	A/C_1_
IT-BG017T	*K. pneumoniae*	AmpAtmCazCroCtx	SHV-12	I1
GR-541	*E. coli*	AmpAtmCazCtxCroFoxIpmTo	VIM-1, CMY13	N
GR-116	*E. coli*	AmpAtmCazCroCtxFoxIpmGmTo	VIM-1, CMY13	N
GR-700	*K. pneumoniae*	AmpCazCroCtxFoxIpmGmTo	VIM-1	N
GR-2564	*K. pneumoniae*	AmpCazCroCtxFoxIpmTo	VIM-1	N
GR-1943	*K. pneumoniae*	AmpCazCroCtxFoxIpmTo	VIM-1	N
GR-1955	*K. pneumoniae*	AmpCazCroCtxFoxIpmTo	VIM-1	N
GR-5866	*K. pneumoniae*	AmpCazCroCtxFoxIpmTo	VIM-1	N
GR-51395	*K. pneumoniae*	AmpCazCroCtxFoxIpmTo	VIM-1	N
GR-6/100	*K. pneumoniae*	AmpCazCroCtxFoxIpmTo	VIM-1	N

*Transconjugants or transformants were obtained by conjugation on solid medium or electroporation, respectively, with *E. coli* (strains DH5-α or DH10B or 26R793 as recipients), as described previously (*5**–**10*). Antimicrobial drug susceptibility of the transconjugants or transformants was determined by disk diffusion as recommended by the Committee on Clinical and Laboratory Standards (*11*) with the following drugs: Amp, ampicillin; Atm, aztreonam; Caz, ceftazidime; Cro, ceftriaxone; Ctx, cefotaxime; Fox, cefoxitin; Ipm, imipenem; Gm, gentamicin; To, tobramycin. Transferred resistance traits were inferred by a detectable reduction of susceptibility to the drug in the transconjugant or transformant, compared to the *E. coli* recipient. †Discrimination between A/C_1_ and A/C_2_ replicons was performed by DNA sequencing of the amplicon obtained by replicon typing.

Eighteen primer pairs were used to perform 5 multiplex and 3 simplex PCRs, which recognized FIA, FIB, FIC, HI1, HI2, I1-Iγ, L/M, N, P, W, T, A/C, K, B/O, X, Y, and FII replicons (*4*). All amplified replicons were sequenced by standard procedures and used as specific probes to confirm the replicon typing results by Southern blot hybridization on purified plasmid DNA (data not shown).

The plasmid donors from the United States consisted of 4 previously characterized ESC-resistant *Salmonella* isolates submitted to the National Antimicrobial Resistance Monitoring System (NARMS) from 1996 to 1998 (*12*) and 6 ESC-resistant *Escherichia coli* O157:H7 isolates collected by NARMS from 2000 to 2001 (*5*). During the study periods, participating state and local public health laboratories forwarded every tenth non-Typhi type *Salmonella* and every fifth *E. coli* O157 isolate they received to the Centers for Disease Control and Prevention for susceptibility testing. This collection includes representatives from sporadic and outbreak infections (*5**,**12*). The 6 *Salmonella* and 4 *E. coli* plasmid donors selected for this study were a small sample of epidemiologically unrelated isolates representative of those carrying a *bla*_CMY-2_ β-lactamase gene on plasmids classified as type A or B on the basis of the *bla*_CMY-2_ hybridization pattern (*6**,**13*). The PCR-based replicon typing method assigned the A/C and I1 replicons to type A and type B plasmids, respectively (Table), which was confirmed by DNA sequencing. The I1-type amplicon sequences were identical to the R64 IncI1 reference plasmid (no. AP005147), whereas the A/C-type amplicon sequences exhibited 26 nucleotide (nt) substitutions with respect to the RA1 IncA/C reference plasmid (no. X73674), which caused 3 amino acid variations. Therefore, the A/C-replicon from the US plasmids may represent a new replicon variant, which we designated repA/C_2_ (DNA sequence released under EMBL accession no. AM087198). The Figure shows conserved *Pst*I restriction profiles obtained for the A/C_2_ plasmids that are different from those exhibited by the I1 plasmids.

The plasmid donors from Italy consisted of 7 multidrug-resistant isolates of various species of *Enterobacteriaceae* carrying either *bla*_SHV-12_ or *bla*_CMY-4_ and *bla*_VIM-4_ plasmidborne β-lactamase genes (Table). These isolates had been collected from 2002 to 2003 at 4 different hospitals in northern or central Italy (*7**,**8*) and were epidemiologically unrelated, except for IT-VA416/02 and IT-VA417/02, which were from the same patient (*7*). PCR replicon typing of the 5 *bla*_SHV-12_–carrying plasmids detected 3 repFII (100% identical to the reference sequence no. M33752), 1 repI1 (100% identical to the R64 plasmid), and 1 repA/C_1_ (99% homologous to the RA1 plasmid) (Table), suggesting mobilization of this gene among different plasmid scaffolds. The *bla*_SHV-12_ plasmids showed different *Pst*I restriction patterns, which confirmed their diversity (Figure). The 2 plasmids carrying *bla*_VIM-4_ and *bla*_CMY-4_ were assigned by PCR replicon typing to the A/C type. The sequence of these replicons showed the same 26 characteristic nucleotide substitutions of the A/C_2_-replicon identified in the US plasmids. These 2 A/C_2_-plasmids showed an apparently identical *Pst*I restriction profile (data not shown), which was also very similar to that of some USA *bla*_CMY-2_ plasmids (see the 2039 and 3977 US plasmids and the Italian VA416/02 plasmid in the Figure). The 2 Italian A/C_2_ plasmids, in addition to *bla*_CMY-4_ (which is a *bla*_CMY-2_ variant different by only a single nucleotide substitution), also carried the *bla*_VIM-4_ carbapenemase gene, which has not been reported on *bla*_CMY-2_–carrying plasmids from the United States and may represent a novel acquisition. These findings indicate intercontinental spread of these plasmids and novel acquisition of resistance genes.

**Figure:**
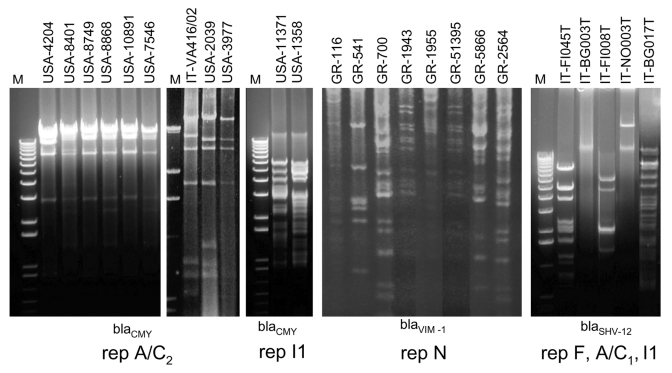
Restriction enzyme analysis of plasmids analyzed in this study. Numbers and letters above each lane indicate strain reference names as defined in the Table. M is the1-kb Plus DNA ladder (Gibco-BRL, Gaithersburg, MD, USA). Plasmids were transferred by conjugation or transformation in *Escherichia coli* K12 strains and purified by Midi-prep purification kit. Plasmids were digested with restriction endonuclease *Pst*I and separated by agarose (1%) gel electrophoresis in 1× TBE buffer. DNA was stained with ethidium bromide and visualized under UV light. The replicons and the β-lactamase genes carried by each group of plasmids are indicated below the figure.

The plasmid donors from Greece consisted of a collection of 7 *Klebsiella pneumoniae* isolates carrying the *bla*_VIM-1_ gene (*9*) and 2 *E. coli* isolates carrying *bla*_VIM-1_ and *bla*_CMY-13_ genes (*10*). These isolates, randomly collected from 5 different hospitals in Athens and Piraeus from 2001 to 2003, are representative of the VIM-1–producing isolates circulating in Greece. No repetitive samples were taken from patients. All isolates exhibited decreased susceptibility to carbapenems. Restriction analysis of these plasmids classified them into 6 different groups on the basis of their restriction profiles (Figure). By replicon typing, all of these plasmids were assigned to the same repN-type replicon, which exhibited 2-nt point mutations (99% homology) in respect to the R46 IncN reference plasmid (no. NC_003292), an indication that they were phylogenetically related and probably evolved from a common ancestor. Although one might expect similar plasmid scaffolds to exist among isolates in Greece and Italy because of geographic proximity, this was not the case. This finding explains the great variability of resistance plasmids carrying different combinations of resistance genes.

Since the origin of replication is a constant and conserved part of a plasmid, replicon typing focused on this portion of the plasmid is a more sensitive and specific method for identifying phylogenetically related plasmids than restriction-based analysis of the entire plasmid. This fact is probably due to the presence of multiple mobile elements (IS elements, transposons, integrons) that can mediate rearrangements of the plasmid scaffolds, which leads to the formation of apparently divergent plasmids. In fact, this phenomenon was demonstrated for the GR-541 plasmid that contains multiple copies of insertion sequences and other mobile genetic elements within its scaffold (*14*).

## Conclusions

A PCR-based replicon typing approach was successfully applied to relevant resistance plasmids. Coupled with sequencing, the approach allowed high-resolution typing of the plasmid replicons. Typing results provided original insights into the molecular epidemiology of resistance plasmids. For instance, the *bla*_CMY-2_–carrying plasmid circulating in the United States was also detected in Europe in the form of a derivative that also carries the VIM-4 carbapenemase determinant. This finding demonstrates that plasmids carrying resistance to clinically relevant antimicrobial agents can spread worldwide among bacteria responsible for both nosocomial and community-acquired infections. The heterogeneity among Italian plasmids encoding SHV-12 (the most prevalent SHV-type extended-spectrum β-lactamase in this country) (*15*) suggests a notable potential for this determinant to spread among different plasmid replicons. On the other hand, replicon typing indicated that the VIM-1–encoding plasmids from Greece were all related despite their different restriction profiles, which points out the common origin of these plasmids. The *bla*_CMY-13_ gene from Greece is located on the repN plasmid, whereas Italy and the United States share the A/C_2_ plasmid as a vehicle of the *bla*_CMY_ gene, despite their geographic distance. Further research is necessary to determine the influences on plasmid trafficking as well as further similarities and differences. Replicon identification may provide useful clues to the evolution of these resistant plasmids. The ability to trace and screen plasmids by PCR may facilitate further understanding of the horizontal transfer of antimicrobial drug resistance.
